# P-288. High Prevalence of Carbapenem-Resistant Gram-Negative Bacteria Among ICU Patients in a Tertiary-Care Hospital

**DOI:** 10.1093/ofid/ofae631.491

**Published:** 2025-01-29

**Authors:** Efthymia Protonotariou, George Meletis, Areti Tychala, Zoe Sereni, Petros Trikoupis, Margarita Oikonomou, Pinelopi Amoiridou, Rizos Rizopoulos, Lampros Tampakas, Parthenopi Pantelidou, Aikaterini Efthymiou, Elena Argiriadou, Paraskevi Mantzana, Lemonia Skoura

**Affiliations:** AHEPA University Hospital, School of Medicine, Aristotle University of Thessaloniki, THESSALONIKI, Thessaloniki, Greece; AHEPA University Hospital, School of Medicine, Aristotle University of Thessaloniki, THESSALONIKI, Thessaloniki, Greece; AHEPA Hospital, Thessaloniki, Thessaloniki, Greece; CLEO, AHEPA University Hospital, Thessaloniki, Thessaloniki, Greece; Cleo, AHEPA Hospital, Thessaloniki, Thessaloniki, Greece; CLEO, AHEPA University Hospital, Thessaloniki, Thessaloniki, Greece; AHEPA University Hospital, School of Medicine, Aristotle University of Thessaloniki, THESSALONIKI, Thessaloniki, Greece; AHEPA University Hospital, School of Medicine, Aristotle University of Thessaloniki, THESSALONIKI, Thessaloniki, Greece; AHEPA University Hospital, School of Medicine, Aristotle University of Thessaloniki, THESSALONIKI, Thessaloniki, Greece; AHEPA University Hospital, School of Medicine, Aristotle University of Thessaloniki, THESSALONIKI, Thessaloniki, Greece; AHEPA University Hospital, School of Medicine, Aristotle University of Thessaloniki, THESSALONIKI, Thessaloniki, Greece; AHEPA University Hospital, School of Medicine, Aristotle University of Thessaloniki, THESSALONIKI, Thessaloniki, Greece; Ahepa University Hospital, Thessaloniki, Thessaloniki, Greece; AHEPA University Hospital, School of Medicine, Aristotle University of Thessaloniki, THESSALONIKI, Thessaloniki, Greece

## Abstract

**Background:**

Infections by carbapenem-resistant organisms (CROs) are causing worldwide concern. Some Gram-negative CROs may act as colonizers of the intestinal flora. Starting from colonization, they may develop serious infections to their host under favorable circumstances. In the present study, the data of patients hospitalized in the Intensive Care Unit (ICU) of AHEPA University Hospital regarding their surveillance rectal detection for carbapenem-resistant Enterobacterales, *Pseudomonas aeruginosa* and *Acinetobacter baumannii* was reviewed.

**Figure:**
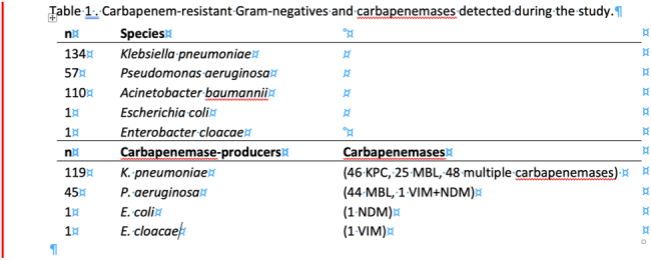

**Methods:**

From October 2022 to December 2023, 578 individual patients’ surveillance rectal specimens were evaluated using the chromogenic Brilliance^TM^CRE Agar (Oxoid, UK). Moreover, among recovered Enterobacterales and *P. aeruginosa* isolates, carbapenemase production was investigated using the NG-Test CARBA 5 multiplex immunoassay (NG-Biotech, France).

**Figure:**
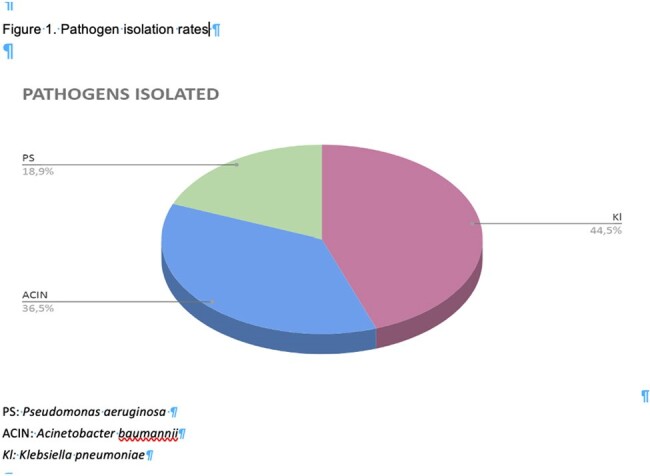

**Results:**

Among the 578 patients evaluated, 178 (30.8%) were colonized with at least one and 68 (38%) with more than one carbapenem-resistant microorganism (Table 1). In total, 303 CROs were identified. *Klebsiella pneumoniae* was present in most samples (n=134); 119/ 134 were found carbapenemase-producers with KPC being the most frequent (n=46) enzyme identified. *A. baumannii* (n=110) followed in order of pathogen detection. 57 carbapenem-resistant *P. aeruginosa* isolates were also recovered and 45 of them were found positive for metallo-β-lactamase production (VIM or NDM) (Figure 1). 115 (64.6%) of the colonized patients were male and 63 (35.4%) were female. Mortality rate for men was 60.87% with an average age of 62 years, while for women it was 72.58% with an average age of 67 years (Figure 2).

**Conclusion:**

Our study highlights the high prevalence of carbapenem-resistant bacteria in ICU patients in our hospital, with a significant proportion of patients being colonized with these organisms. Additionally, the identification of the most common carbapenemase-producing pathogens as well as the high mortality rates among colonized patients provides valuable information for guiding treatment strategies and improving patient outcomes.

**Disclosures:**

**All Authors**: No reported disclosures

